# What does women’s empowerment have to do with malnutrition in Sub-Saharan Africa? Evidence from demographic and health surveys from 30 countries

**DOI:** 10.1186/s41256-019-0129-8

**Published:** 2020-01-14

**Authors:** Sanni Yaya, Emmanuel Kolawole Odusina, Olalekan A. Uthman, Ghose Bishwajit

**Affiliations:** 1 School of International Development and Global Studies, University of Ottawa, 120, University Private, Ottawa, ON K1N 6N5 Canada; 2 The George Institute for Global Health, The University of Oxford, Oxford, UK; 3 2Department of Demography and Social Statistics, Federal University, Oye, Ekiti Nigeria; 4 Warwick Centre for Applied Health Research and Delivery (WCAHRD), Division of Health Sciences, Warwick Medical School, University of Warwick, Coventry, CV4 7AL UK

**Keywords:** Women’s empowerment, Stunting, Underweight, Childhood nutrition, Global health, Sub Saharan Africa

## Abstract

**Background:**

The reduction of childhood malnutrition has been identified as a priority for health and development in sub Saharan African countries. The association between women’s empowerment and children’s nutritional status is of policy interest due to its effect on human development, labour supply, productivity, economic growth and development. This study aimed to determine the association between women’s empowerment and childhood nutritional status in sub Saharan African countries.

**Methods:**

The study utilized secondary datasets of women in their child bearing age (15–49 years) from the latest Demographic and Health Survey (DHS) conducted in 2011–2017 across 30 sub Saharan Africa countries. The outcome variable of the study was childhood nutritional status while the exposure variable was women’s empowerment indicators such as decision making and attitude towards violence. Analyses were performed at bivariate level with the use of chi square to determine association between outcome and exposure variables and at multivariate level with the use of regression models to examine the effect of women’s empowerment on childhood nutritional status.

**Results:**

Women’s socio-demographic and other selected characteristics were statistically significantly associated with childhood nutritional status (stunted and underweight) at *p* < 0.001. These characteristics were also statistically significantly associated with empowerment status of women (Decision-making, Violence attitudes and Experience of violence) at p < 0.001 except for child age and sex. The association between childhood nutritional statuses and women’s empowerment (all three empowerment measures) was significant after controlling for other covariates that could also influence childhood nutrition statuses at *p* < 001. Two of the empowerment measures (attitudes towards violence and experience of violence) showed positive association with childhood nutritional statuses while the third (decision-making) showed negative association.

**Conclusion:**

There is an independent relationship between childhood nutrition status and women’s empowerment in sub Saharan African countries. Women’s empowerment was found to be related to childhood nutritional status. Policies and programmes aiming at reducing childhood malnutrition should include interventions designed to empower women in Sub-Saharan Africa.

## Background

In spite of the worldwide improvement in nutritional status of children [1–3] especially in developing countries [4, 5], malnutrition in childhood is still a major health challenge in low income countries [6] and also in the world at large [1, 7]. Child under nutrition is a factor in more than three million preventable childhood deaths. Estimation indicated about 165 million under 5 children are stunted while wasting accounted for 52 million [1, 2]. Globally, 45% of child mortality is due to malnutrition [8, 9]. In 2015, estimates put the number of child mortality due to malnutrition at 405700 [1, 8]. Due to persisting malnutrition, the World Health Organisation called for worldwide action to stem down the tide of stunting among children by 40% by 2025 [8, 9].

Malnutrition contributes substantially to childhood mortality and morbidity in sub-Saharan African countries [10]. Sub-Saharan countries are notable for one of the worst cases of wasting among children, low level of birth weight and underweight. The state of malnutrition in sub Saharan Africa is alarming and requires urgent policies and programmes to alleviate the scourge [11]. In addition, among 162 million stunted under five children, 36% resided in Africa. In fact, Black et al. [1] and Na, Jennings, Talegawkar and Ahmed [3] put the figure at approximately 40%.

Overall, developing countries had an estimated 60 million under five stunted children [12]. it was also observed that wasting increased from 11% in 2003 to 18% in 2013, underweight from 24% in 2003 to 29% in 2013 while stunted decrease from 42% in 2003 to 37% in 2013 [12]. The rate of malnutrition is a serious source of concern and also due to its potential effect in the later years of children. Early childhood malnutrition could be a potential cause of low cognitive skills, and resultant low productivity and income-earning ability, vulnerability to chronic diseases and obesity when adult age is attained [13–21]. Therefore, reduction in malnutrition can be of health and economic gain. Existing evidence on factors associated with malnutrition and child health in low income countries emphasised cultural, economic, social and/or infrastructural factors at community, household and individual levels [13, 22–24]. Towards findings determinants of childhood health and malnutrition, studies have also focus on many variables such as domestic violence, maternal education and other maternal characteristics [13, 22, 24, 25]. Of all the factors associated with childhood health and malnutrition, contextually, maternal characteristics such as age, literacy, employment, income and education have been found to be significant [13, 25, 26].

Evidences revealed studies on relationship between women’s empowerment and childhood malnutrition [2, 3, 27, 28]. However, more needs to be done in sub Saharan African countries to identify factors and conditions promoting incidence of childhood malnutrition in the region due to high level of childhood malnutrition. An effort towards eliminating childhood malnutrition worldwide would consider sub Saharan Africa as a strong study area to know the associated factors, principal causes and solutions. In addition, data on malnutrition among children in Africa are limited [29]. Though study revealed female labour force participation was not related to malnutrition after controlling for economic development and that women’s empowerment and wellbeing might not be a function of women’s employment [30]. Literature advocated women’s empowerment as a means of improving nutritional status of children. Women empowerment being a concept with multi-dimensional measurement [13, 25] and its association with childhood malnutrition has not being adequately explored.

Empowerment as a concept has also been given multidimensional explanations and features [13, 31, 32]. Among many explanations of the concept is the assertion that empowerment entails access, capability to make choice and control over resources [32] while Malhotra, Schuler and Boender argued that patriarchal systems transformation is a prerequisite for achievement of women empowerment [33]. As put forward by Kabeer [32], empowerment connotes a process of change and ability to exercise choice in one’s life by people who had earlier on deprived of such opportunities. She opined that there was association between disempowerment and poverty. People with limited resources may find it difficult to exercise meaningful choice, though not all choices are equally relevant to empowerment. She looked at ability to exercise choice from three interrelated ways (resources, agency and achievements). By resources she referred to material, social and human resources that facilitate the capacity to make choice and this can be acquired through social relationship in different institutions of the society. People are endowed with ability to make choice due to their position within the society. Agency refers to ability to recognise and the capacity to pursue one’s life goals despite opposition from others. It also refers to capacity to override other through coercion, threat and violence. Sen explained the two terms (resources and violence) as the potential to live according to one’s desire and to achieve valued ways of living recognised and valued in the society. He called them ‘functioning achievements’, all ways of life valued by the society [34]. However, Kabeer [32] reiterated that the three dimensions of empowerment are indivisible. As evident from literature, this study examined three domains of women empowerment and their association with childhood nutritional status [31, 35]. The ability of women to participate in decision making especially in line with resource control and their attitude and experience of violence may affect their lives and also reflect on the nutritional status of children. Women empowerment in the past had been measured using proxies like women’s education and labour force participation rate which only reflect access to resources but not ability to control or take decision as regard the resources [36].

This study considers datasets from 30 sub-Saharan African countries to do in-depth statistical analysis of women’s empowerment and its association with childhood nutritional status. It assesses the relationship between women’s empowerment and childhood nutritional status as well as other predictors of childhood malnutrition in the continent.

## Methods

### Study design and data source

This cross-sectional study used data collected from women (15–49 years) of reproductive ages from the latest Demographic and Health Surveys (DHS) conducted between 2011 and 2017 across 30 countries of sub Saharan Africa (see Table 1 for survey characteristics). DHS was designed to collect and provide data on demographic, fertility and family planning as well as information necessary to monitor population health indicators and vital statistics. Demographic and Health Surveys (DHS) are national and representative with special interest on reproductive health, child health, fertility, nutrition, mortality and health behaviour [37]. Data collected through DHS are robust, help in health research and are used to study and monitor prevalence, pattern and trends. To select the sample, multi-stage stratified cluster sampling method was employed and the eligible respondents were selected from rural and urban areas in the country. Data were related to households, women, men, couples and children were collected by means of different questionnaires. Standard methods were employed to test the validity and reliability of the questionnaires. Information, scope and other related concepts of the Demographic and Health Surveys (DHS) are well documented [38]. Demographic and health Surveys (DHS) datasets are available for researchers through DHS at http://dhsprogram.com/data/available-datasets.cfm.

**Table 1:** Survey characteristics

Country	Survey year	N
Benin	2016–17	5427
Burundi	2016–17	5600
Cameroon	2011	4167
Chad	2014–15	7910
Comoros	2012	1971
Cote d’Ivoire	2012	2225
Democratic Republic of Congo	2013–14	6320
Ethiopia	2016	8780
Gabon	2012	1690
Gambia	2013	2547
Ghana	2014	2256
Guinea	2012	2797
Kenya	2014	6728
Lesotho	2014	850
Liberia	2013	1990
Malawi	2015–16	4246
Mali	2013	4049
Mozambique	2011	7591
Namibia	2013	620
Niger	2012	4752
Nigeria	2013	22,669
Rwanda	2014–15	2946
Senegal	2011	2804
Sierra Leone	2013	3386
South Africa	2016	402
Tanzania	2015–16	7198
Togo	2013–14	2674
Uganda	2016	3568
Zambia	2013–14	8681
Zimbabwe	2015	4432
Total		141,275

Determinants of childhood nutritional status for under-five children in sub Saharan African countries were estimated by focusing on women’s empowerment. Women’s empowerment was measured by household decision-making index, attitude towards violence index and lifetime experience of violence index.

### Variables measurement

#### Nutritional status of children measurement

To provide answer to the question ‘What is the extent of relationship between women’s empowerment and children under-5 nutritional status in sub-Saharan African countries?’, dataset from 30 sub-Saharan countries with information on nutritional status of children were considered. Positive relationship is expected between women’s empowerment and children nutritional status. Improvement in women’s empowerment is expected to lead to well-being of children inform of reduction in their nutritional status. This involves height and weight measurement of children under-5; height-for-age (HAZ) and weight-for-age (WAZ) were used to measure stunting and underweight respectively. Using WHO Child Growth Standards as the reference population, the indices encompassed z-scores of standard deviation (SD) units from the median of the reference population. HAZ reflects linear growth and chronic undernutrition in early childhood (stunting) while WAZ reflects both acute and chronic undernutrition (underweight). The − 2 standard deviations from the reference population median represent the cut-off point for WAZ (underweight) and HAZ (stunting). Children having z-scores greater than − 2.00 were coded as not stunted/underweight, that is 0, while children having z-scores of less or equal to − 2.00 were coded as stunted/underweight, that is 1.

#### Women’s empowerment measurement

In this study, three quantitative variables (as evident from literature) represent women’s empowerment: household decision-making index, attitude towards violence index and lifetime experience of violence index [31, 35]. Recent studies on nutritional status of children and women’s empowerment used simple additive index [31, 35, 39]. To align with these studies, simple additive index was created for decision-making and attitudes towards violence variables. Regression models were fitted and other determinants of women’s empowerment and children’s nutritional statuses were controlled to estimate the influence of women’s empowerment on child malnutrition. Further analysis explored the association with other factors.

#### Other covariates

Variables that were identified as covariates in previous studies on women’s empowerment and child undernutrition were explored as potential risk factors, confounders or effect modifiers [31, 35, 40, 41]. The following variables were explored as covariates: household wealth (poorest, poorer, middle, richer, and richest), place of residence (rural/urban), region, child’s age (months), child’s sex (male/female), child’s birth order (1st-2nd = 1, 3rd-4th = 2, 5th–6th = 3, >6th = 4), respondent’s age (years), respondent’s Body Mass Index (low BMI = < 18.5 kg/m2), respondent’s and respondent’s husband’s education (no education = 0, primary = 1, secondary = 2, higher = 3), respondent and husband’s age differential (husband older = 0; respondent same age or older = 1), respondent and husband’s education differential (women has less education = 0; woman has more education = 1) and age at first marriage (years).

#### Ethical consideration

Secondary data, Demographic and Health Survey (DHS) datasets were used. Before the surveys were carried out, ethical clearance was obtained from the ethical committees of the respective countries. In addition, before participation, informed consent was also obtained from the women. Moreover, all DHS are approved by ICF international and Institutional Review Board (IRB) to ensure that the protocols comply with the U.S. Department of Health and Human Services regulations for the protection of human subjects. The data from the surveys were completely anonymized.

#### Data analysis

The study used Demographic and Health Surveys from 30 sub-Saharan African countries to examine women’s empowerment and nutritional status of children. The most recent survey conducted by each country was considered. Sampling weights were accounted for. Being national surveys, weights were applied because the response rates vary among the different population groups or secondary sampling units. The sampling weights or mathematical adjustments were also applied to the data to correct for under-sampling and oversampling. Data were examined at univariate, bivariate and multivariate levels. At univariate level of data analysis, frequency and percentage distributions of variables were employed to describe outcomes, exposures and other characteristics of respondents. At bivariate level, associations between outcomes, exposure (experience of violence) and other covariates were explored with the use of chi square tests. In addition, Ranksum and Kruskal Wallis tests were used to examine the association with employment exposures (attitude towards violence and decision-making). The test of correlation between variables revealed assumptions of multicollinearity were not violated. At multivariate level, regression model was employed. A regression model was fitted to investigate independent effect of women’s empowerment on nutritional status of children after controlling for respondents’ age, BMI and education, child’s sex and age, household wealth and area of residence. In addition, the study considers similarity of the results by key demographic characteristics. Age is an important demographic variable. It can affect reproductive behaviour and other characteristics of respondents and that of their partners. Age of respondents may be related to characteristics such as education, BMI and child age. Therefore, further analyses involved interaction with age difference between respondents and interaction with age difference between partners while respondents’ age, BMI and education, child’s sex and age, household wealth and area of residence where adjusted.

## Results

The women’s empowerment and childhood nutritional status by country are presented in Table 2. There were variations between countries in sub-Saharan Africa.

**Table 2:** Women’s empowerment and nutritional status by country

Variables/ Country	Stunted Cases (%)	Underweight Cases (%)	Involvement in decision-making (No) Cases (%)	Attitude to violence (In support) Cases (%)	Experience of violence (Yes) Cases (%)
Benin	1746 (32.2%)	926 (17.1%)	1589 (29.3%)	1785 (32.9%)	387 (7.1%)
Burundi	3103 (55.4%)	1609 (28.7%)	809 (14.4%)	3366 (60.1%)	696 (12.4%)
Cameroon	1347 (32.3%)	618 (14.8%)	1300 (31.2%)	1957 (47.0%)	287 (6.9%)
Chad	3192 (40.4%)	2388 (30.2%)	3238 (40.9%)	6255 (79.1%)	2, 576 (32.6%)
Comoros	588 (29.8%)	304 (15.4%)	825 (41.9%)	827 (42.0%)	176 (9.0%)
Cote d’Ivoire	639 (28.7%)	322 (14.5%)	982 (44.1%)	1140 (51.3%)	258 (11.6%)
Democratic Republic of Congo	2720 (43.0%)	1422 (22.5%)	1671 (26.4%)	4847 (76.7%)	1089 (17.2%)
Ethiopia	3350 (38.2%)	2056 (23.4%)	1045 (11.9%)	6027 (68.7%)	2327 (26.5%)
Gabon	260 (15.4%)	88 (5.2%)	219 (13.0%)	938 (55.5%)	91 (5.4%)
Gambia	623 (24.5%)	423 (16.6%)	422 (16.6%)	1651 (64.8%)	266 (10.4%)
Ghana	397 (17.6%)	242 (10.7%)	185 (8.2%)	760 (33.7%)	138 (6.1%)
Guinea	861 (30.8%)	501 (17.9%)	1149 (41.1%)	2634 (94.2%)	1272 (45.5%)
Kenya	1720 (25.6%)	730 (10.9%)	802 (11.9%)	3100 (46.1%)	318 (4.7%)
Lesotho	245 (28.8%)	81 (9.6%)	22 (2.6%)	297 (35.0%)	23 (2.7%)
Liberia	602 (30.2%)	274 (13.8%)	167 8.4%)	933 (46.9%)	73 (3.7%)
Malawi	1543 (36.3%)	468.8 (11.0%)	604 (14.2%)	658 (15.5%)	76 (1.8%)
Mali	1573 (38.9%)	1028 (25.4%)	2965 (73.2%)	3229 (79.7%)	808 (20.0%)
Mozambique	3226 (42.5%)	1137 (12.9%)	1046 (13.8%)	1785 (23.5%)	134 (1.8%)
Namibia	123 (19.9%)	80 (12.9)	33 (5.4%)	208 (33.5%)	45 (7.3%)
Niger	2041 (42.9%)	1, 716 (36.1%)	2850 (60.0%)	2914 (61.3%)	1448 (30.5%)
Nigeria	8427 (37.2%)	6604 (29.1%)	11,312 (49.9%)	8654 (38.2%)	2798 (12.3%)
Rwanda	1084 (36.8%)	243 (8.2%)	210 (7.1%)	1187 (40.3%)	129 (4.4%)
Senegal	773 (27.6%)	504 (18.0%)	1524 (54.4%)	1847 (65.9%)	626 (22.3%)
Sierra Leone	1274 (37.6%)	538 (15.9%)	1082 (32.0%)	2446 (72.3%)	625 (18.4%)
South Africa	101 (25.2%)	13 (3.2%)	10 (2.6%)	26 (6.6)	00 (00.0%)
Tanzania	2437 (33.9%)	976 (13.6%)	1411 (19.6%)	4440 (61.7%)	1262 (17.5%)
Togo	700 (26.2%)	412 (15.4%)	742 (27.7%)	875 (32.7%)	166 (6.2%)
Uganda	1007 (28.2%)	349 (9.8%)	520 (14.6%)	1757 (49.2%)	211 (5.9%)
Zambia	3463 (39.9%)	1239 (14.3%)	1118 (12.9%)	4.452 (51.3%)	1515 (17.5%)
Zimbabwe	1127 (25.4%)	334 (7.5%)	170 (3.8%)	1734 (39.1%)	192 (4.3%)
Total	50,291 (35.6%)	27,623 (19.6%)	40,025 (28.3%)	72,730 (51.5%)	20, 012 (14.2%)

The frequency and percentage distribution of socio-demographic characteristics of respondents (women in their reproductive ages) were explored for outcome variables. As Table 3 revealed, about a quarter of the total respondents (22.7%) and (22.2%) belonged to poorest and poorer wealth index categories. Each of the other categories of wealth index had less than one-fifth of the total respondents (middle, 19.9%, richer, 18.9% and richest, 16.3%). Majority of the respondents (71.8% versus 28.2%) indicated they resided in rural areas. The age distributions of children were about one-fifth for each age category and sex distributions of children were also about the same (male, 50.1% versus female, 49.9%). The percentage distribution of birth order followed a descending pattern, the higher the percentage, the lower the birth order. By age distribution of respondents, it followed an ascending order on till 30–34 age group while descending pattern started from 35 to 39 age group and continued till 45–49 age group. Respondents without low BMI constituted 90.7% of the total respondents while 9.3% reported low BMI. Distribution by educational attainment revealed a descending pattern - 42.2% had no formal education, 34.7% had primary education, 20.0% had secondary education and 3.1% had higher level of education.

**Table 3:** Relationship between outcomes and Exposures with Covariates

Covariates			Stunted		Underweight		Decision-making	Violence Attitudes	Experience of violence
Variable	Category	Frequency (%)	Chi-2 *p*-value	Cases (%)	Chi-2 *p*-value	Cases (%)	Rank sum/Kruskil Wallis *p*-value		Chi-2 *p*-value
Wealth Index	Poorest	32,073 (22.7)	< 0.001	14,213 (44.3)	< 0.001	8363 (26.1)	< 0.001	< 0.001	< 0.001
	Poorer	31,291 (22.2)		12,861 (41.1)		7095 (22.7)			
	Middle	28,160 (19.9)		10,256 (36.4)		5292 (18.8)			
	Richer	26,679 (18.9)		8284 (31.1)		4364 (16.4)			
	Richest	23,072 (16.3)		4677 (20.3)		2509 (10.9)			
	Total	141,275 (100)		50,291 (35.6)		27,623 (19.6)			
Type of Residence	Urban	39,889 (28.2)	< 0.001	9988 (25.0)	< 0.001	5405 (13.6)	< 0.001	< 0.001	< 0.001
	Rural	101,386 (71.8)		40,303 (39.8)		22,218 (21.9)			
	Total	141,275 (100)		50,291 (35.6)		27,623 (19.6)			
Child age	< 1 year	30,188 (21.4)	< 0.001	5518 (18.3)	< 0.001	4417 (14.6)	< 0.001	0.822	0.180
	1	29,238 (20.7)		11,222 (38.4)		6110 (20.9)			
	2	27,362 (19.4)		12,274 (44.9)		5926 (21.7)			
	3	27,736 (19.6)		11,561 (41.7)		5695 (20.5)			
	4	26,752 (18.9)		9716 (36.3)		5476 (20.5)			
	Total	141,275 (100)		50,291 (35.6)		27,623 (19.6)			
Sex of Child	Male	70,839 (50.1)	< 0.001	26,709 (37.7)	< 0.001	14,658 (20.7)	0.601	1.000	0.859
	Female	70,436 (49.9)		23,582 (33.5)		12,965 (18.4)			
	Total	141,275 (100)		50,291 (35.6)		27,623 (19.6)			
Birth order	1st-2nd	51,827 (36.7)	< 0.001	17,402 (33.6)	< 0.001	8871 (17.1)	< 0.001	< 0.001	< 0.001
	3rd-4th	42,151 (29.8)		14,651 (34.8)		7958 (18.9)			
	5th–6th	26,607 (18.8)		10,059 (37.8)		5727 (21.5)			
	More than 6th	20,691 (14.7)		8180 (39.5)		5067 (24.5)			
	Total	141,275 (100)		50,291 (35.6)		27,623 (19.6)			
Respondents’ age	15–19	6541 (4.6)	< 0.001	2419 (37.0)	< 0.001	1368 (20.9)	< 0.001	< 0.001	< 0.001
	20–24	28,864 (20.4)		10,739 (37.2)		5562 (19.3)			
	25–29	39,985 (28.3)		14,028 (35.1)		7610 (19.0)			
	30–34	31,566 (22.3)		10,738 (34.0)		5957 (18.9)			
	35–39	21,410 (15.2)		7545 (35.2)		4391 (20.5)			
	40–44	9859 (7.0)		3629 (36.8)		2040 (20.7)			
	45–49	3050 (2.2)		1193 (39.1)		695 (22.8)			
	Total	141,275 (100)		50,291 (35.6)		27,623 (19.6)			
Maternal BMI	Not low BMI	128,170 (90.7)	< 0.001	44,510 (34.7)	< 0.001	23,280 (18.2)	< 0.001	< 0.001	< 0.001
	Low BMI	13,106 (9.3)		5781 (44.1)		4343 (33.1)			
	Total	141,275 (100)		50,291 (35.6)		27,623 (19.6)			
Educational attainment	No education	59,627 (42.2)	< 0.001	25,153 (42.2)	< 0.001	16,293 (27.3)	< 0.001	< 0.001	< 0.001
	Primary	49,037 (34.7)		17,789 (36.3)		7853 (16.0)			
	Secondary	28,302 (20.0)		6824 (24.1)		3207 (11.3)			
	Higher	4309 (3.1)		525 (12.2)		270 (6.3)			
	Total	141,275 (100)		50,291 (35.6)		27,623 (19.6)			
Respondent and husband age differential	> 8 years	52,856 (37.4)	< 0.001	19,089 (36.1)	< 0.001	12,045 (22.8)	< 0.001	< 0.001	< 0.001
	8–4 years	53,483 (37.9)		18,667 (34.9)		9841 (18.4)			
	3-1 years	30,733 (21.8)		10,979 (35.7)		5016 (16.3)			
	No diff or older	4203 (3.0)		1556 (37.0)		721 (17.2)			
	Total	141,275 (100)		50,291 (35.6)		27,623 (19.6)			
Respondent and husband educational differential	Same Education	57,186 (40.5)	< 0.001	22,207 (38.8)	< 0.001	14,190 (24.8)	< 0.001	< 0.001	< 0.001
	Less education	60,565 (42.9)		20,482 (33.8)		9966 (16.5)			
	More education	23,524 (16.7)		7602 (32.3)		3467 (14.7)			
	Total	141,275 (100)		50,291 (35.6)		27,623 (19.6)			
Husbands’ education	No education	48,002 (34.0)	< 0.001	20,414 (42.5)	< 0.001	13,566 (28.3)	< 0.001	< 0.001	< 0.001
	Primary	46,178 (32.7)		17,268 (37.4)		7905 (17.1)			
	Secondary	37,937 (26.9)		10,936 (28.8)		5226 (13.8)			
	Higher	9158 (6.5)		1672 (18.3)		926 (10.1)			
	Total	141,275 (100)		50,291 (35.6)		27,623 (19.6)			
Age at first marriage	15–17 years	50,690 (35.9)		19,937 (39.3)	< 0.001	11,372 (22.4)	< 0.001	< 0.001	< 0.001
	18–20 years	48,377 (34.2)		17,318 (35.8)		9114 (18.8)			
	21–23 years	24,770 (17.5)		8107 (32.7)		4282 (17.3)			
	24–26 years	10,586 (7.5)		3157 (29.8)		1789 (16.9)			
	27 plus	6852 (4.9)		1773 (25.9)		1065 (15.5)			
	Total	141,275 (100)		50,291 (35.6)		27,623 (19.6)			

Percentage distribution by age differential showed that 37.4% of respondents were more than eight years younger than their husbands while 37.9% indicated they were eight to four years younger than their husbands. In addition, those who were three to one year younger than their husbands were 21.8%, those who indicated no age difference or older than their husband were less than 5% (3.0%). Distribution by educational difference revealed 40.5% had the same education, 42.9% less educated than their husband and 16.7% more educated than their husbands. More than one-third (35.9%) of the respondents married at 15–17 years age range and also more than one-third (34.2%) married at 18–20 years age range while 29.9% married at later ages.

As indicated in Table 3, all the socio-demographic and other selected characteristics of respondents were statistically significantly associated with childhood nutritional status (stunted and underweight) at *p* < 0.001. These characteristics were also statistically significantly associated with empowerment status of women (Decision-making, Violence attitudes and Experience of violence) at p < 0.001 except for child age and sex (see Table 2). Table 4 revealed correlation among the covariates. The correlation between covariates was less than 0.7. Moreover, correlation between main exposure variables was less than 0.13 except between attitudes towards violence and experience of violence which was 0.72, therefore, the two variables were not considered in the same analysis (Table 5).

**Table 4:** Correlation between Covariates

Correlation (*p*-value)	Respondents’ age	Respondents’ education	Maternal BMI	Child age	Husband’s education	Age differential	Wealth
Respondents’ age	1.0000						
Respondents’ education	−0.1003 (< 0.001)	1.0000					
Maternal BMI	0.0317 (0.0000)	0.1126 (< 0.001)	1.0000				
Child age	0.1801 (< 0.001)	−0.0422 (< 0.001)	0.0084 (0.002)	1.0000			
Husband’s education	−0.0686 (< 0.001)	0.6465 (< 0.001)	0.1107 (< 0.001)	−0.0312 (< 0.001)	1.0000		
Age differential	0.0377 (< 0.001)	0.1445 (< 0.001)	0.0263 (< 0.001)	− 0.0115 (< 0.001)	0.1338 (< 0.001)	1.0000	
Wealth	0.0103 (< 0.001)	0.4107 (< 0.001)	0.1071 (< 0.001)	0.0001 (< 0.9672)	0.3879 (< 0.001)	0.0103 (< 0.001)	1.0000

**Table 5:** Correlation between Main Exposure Variables

Correlation (*p*-value)	Decision-making	Attitudes towards violence	Experience of violence
Decision-making	1.0000		
Attitudes towards violence	−0.1701 (< 0.001)	1.0000	
Experience of violence	−0.1248 (< 0.001)	0.7182 (< 0.001)	1.0000

Results from Table 6 revealed the result of multivariate analyses. The association between childhood nutritional statuses and women’s empowerment (all three empowerment measures) was significant after controlling for other covariates that could also influence childhood nutrition statuses. Two of the empowerment measures (attitudes towards violence and experience of violence) showed positive association with childhood nutritional statuses while the third (decision-making) showed negative association. The study showed the existence of independent relationship between childhood nutrition statuses and women’s empowerment in sub Saharan African countries. However, the association did not imply causality. This study cannot assert causality because the data used were cross-sectional. In addition, the association between childhood nutritional statuses and women’s empowerment was also examined with age of respondents and age difference between partners as interactive factors. The results revealed large variability. However, it can be established that women’s empowerment was associated with childhood nutritional statuses due to evident of statistically significant relationship.

**Table 6:** Fully adjusted Model and Model with Interaction

		Full adjustment*			Interaction with age difference between partners					
Variable	Category/unit	Stunting			Respondent younger than husband or no difference			Respondent older than husband		
		OR	95% CI	*p*-value	OR	95% CI	*p*-value	OR	95% CI	*p*-value
Decision-making		0.84	0.83, 0.86	0.001	0.88	0.77, 1.01	0.08	1.13	0.99, 1.30	0.08
Any Experience of violence		1.07	1.04,1.11	0.001	0.91	0.74, 1.12	0.34	1.10	0.89, 1.36	0.38
					Interaction with Age of Respondent					
Variable	Category/unit	Underweight			Respondent Age 15–29			Respondent Age 30–49		
Attitude towards violence		1.13	1.10, 1.16	0.001	0.98	0.93, 1.04	0.52	1.02	0.96, 1.08	0.52

*Adjusted for respondents’ age, BMI and education, child’s sex and age, household wealth and area of residence

### Discussion and policy implications

The study reported more respondents in the poorest and poorer wealth index categories compared to richer and richest categories. Income has been found to be associated with women’s empowerment. Having more of the respondents in the poorest and poorer wealth index categories was a pointer to low women’s self-esteem and therefore low women’s empowerment [13, 25, 26]. This corroborated Kabeer’s theory on empowerment and poverty. Inability of people to exercise choice may result due to limited resources. She associated poverty with low women’s empowerment [32]. Wealth index as a factor was found to be associated with women’s empowerment. This had semblance with the fact that majority of the respondents also lived in rural areas. About half of them had no formal education and more than seven in ten married before age 20. Low level of education and early marriage may also lead to low self-esteem which may subsequently hamper women’s empowerment [13, 25, 26]. It was also noted that the same proportion of respondents had less than one, one, two, three- and four-years old children. These were not their completed fertility. In addition, sex distribution followed similar pattern with male and female having about the same proportion. Birth order of respondents revealed those with one to two children had the highest proportion while lowest proportion was from respondents with six or more children.

It should be noted that many of the respondents with one to two children would have more children because they had not attained their completed fertility. More than one-third of respondents belonged to 20–34 age range while about one-tenth of the total respondents had low BMI. Very few women (respondents) reported that they were of equal age or older than their husbands while about 2 in 5 had the same education or less educated compared to their husbands. Moreover, altogether, about six in ten women (respondents) were less educated than their husbands. Women’s education enhances women’s empowerment and their participation in decision-making [13, 22–24]. Disparity and low educational attainment on the part of women may influence their participation in decision-making and empowerment.

The study reported disparity in childhood nutritional status by socio-demographic characteristics. This might not be unconnected with disparities in educational attainment, place of residence and other factors. Though, these socio-demographic variables were related to childhood nutritional status [22–24, 31]. Women BMI was found to be significantly associated with nutritional status of their children. Indeed, the well-being of mothers may translate or reflect in the well-being of their children. Empowered women may have more potential to meet the needs of their children compared to their counterparts. Moreover, the distribution of women’s empowerment measures showed inequalities across socio-demographic and other selected characteristics. Significant associations were observed between women’s empowerment measures and socio-demographic and other selected characteristics except for age and sex of children. Multivariate regression model fitted to investigate independent effect of women’s empowerment measures on nutritional status of children revealed women’s empowerment was independently related to childhood nutritional status. Findings from this study were consistent with the previous studies that have shown a relationship between women empowerment and child health outcomes [31, 42]. The results from this study suggest that women’s empowerment is an incredibly complex issue, and that childhood welfare and nutritional status are linked to the degree of autonomy the woman has in effecting change in her household.

Childhood malnutrition affects in sub-Saharan Africa affects more than 1 in 3 children and although child nutritional stats have improved over the past several decades, countries continue to struggle in the fight against malnutrition [40]. The Sustainable Development Goals (SDGs) aim to end hunger, achieve food security, improve nutrition especially among children and the most vulnerable by 2030. This study thus shows that in order to achieve nutrition justice, women’s status in terms of the three dimensions (household decision-making index, attitude towards violence index and lifetime experience of violence index) should be taken into consideration in interventions by policymakers as well as international bodies. These interventions should be gender-responsive so to help transform norms, stereotypes and attitudes that uphold female subordination,

### Strengths and limitations

The datasets are not only from many countries but also nationally representative. In fact, the sample size collected from these rounds of surveys was sufficiently large and by standard procedure which increases the external validity of the findings for women aged between 15 to 49 years in Sub-Saharan Africa. However, there are also limitations to the study. Other studies [43–45] for instance have shown that religion is associated with women’s empowerment and that “any meaningful effort to promote women empowerment in Africa must account for the continent’s three main religions” [43]. However, religion as a variable was not considered in our study because information on religion is not representative due to a lot of missing values in the dataset. In addition, the surveys used cross-sectional design which only permits associations but not causalities. Moreover, DHS surveys were conducted in different years, comparison of results from different surveys should be done with caution [46, 47].

## Conclusion

The study explored the association between women’s empowerment and childhood nutritional status in sub Saharan African countries. Despite the efforts at reducing childhood malnutrition statuses, the persistent burden of malnutrition among children threatens the health and future development in the region and also the achievement of Sustainable Development Goals for child and maternal health. To reduce childhood malnutrition and prevent the associated ill-health, risk factor such as women’s empowerment needs to be addressed. Necessary policies, programmes and interventions channelled towards women’s empowerment could help to reduce childhood malnutrition. Moreover, childhood malnutrition could be addressed at the population and individual levels taking cognizance of the factors associated with women’s empowerment.

## Data Availability

Data for this study were from Demographic and Health surveys (DHS) and available here: http://dhsprogram.com/data/available-datasets.cfm.
